# The first complete mitochondrial genome from the subfamily Phyllocephalinae (Heteroptera: Pentatomidae) and its phylogenetic analysis

**DOI:** 10.1080/23802359.2017.1413313

**Published:** 2017-12-11

**Authors:** Chao Chen, Jiufeng Wei, Wei Ji, Qing Zhao

**Affiliations:** a Department of Environment Science and Engineering, Taiyuan University of Technology, Taiyuan, Shanxi, PR China;; b Department of Entomology, Shanxi Agricultural University, Taigu, PR China;; c College of Horticulture, Shanxi Agricultural University, Taigu, Shanxi, PR China

**Keywords:** Heteroptera, Phyllocephalinae, *Gonopsis affinis*, mitogenome, phylogeny

## Abstract

The true bug, *Gonopsis affinis*, is an important pest in China. Here, we determined the complete mitogenome of *G. affinis*, which is the first for the subfamily Phyllocephalinae. This 16,011-basepair (bp) mitogenome comprises of 13 protein-coding genes (PCGs), 22 transfer RNA genes (tRNAs), two ribosomal RNA genes, and an A + T-rich region. The gene order and the orientation are similar to those of other sequenced Hemipteran species. All PCGs start with ATN codons, except *COI*, and end with TAA. The 22 tRNAs have a typical cloverleaf secondary structure except trnS-*Ser* ^(AGN)^, which lacks a dihydrouridine (DHU) arm. Phylogenetic analyses highly supported the monophyly of each family, and confirmed the reasonable placement of *G. affinis*.

Phyllocephalinae (Heteroptera: Pentatomomorpha), a subfamily of Pentatomidae, is phytophagous and causes damage to agriculture and forest. Despite this destructive feeding behaviour, genetic information for this species is needed. To date, the pentatomid mitogenomes have been obtained from Pentatominae (Zhao et al. 2017a), Podopinae (Wang et al. 2017) and Asopinae (Zhao et al. 2017b), which limits our understanding of the diversity and phylogeny of Pentatomidae. In the following, we report and analyze the complete mitogenome of *Gonopsis affinis* and provide molecular and phylogenetic information for studies on Pentatomoidea of Hemiptera.

Adult specimens of *G. affinis* were collected from Xiaguan (25.58 N, 100.23 E), Dali City, Yunnan Province, China, on 18 August 2015. Voucher specimens (No. SXAU2017002) and remaining genomic DNA were deposited in the Institute of Entomology, Shanxi Agricultural University, Taigu, China. This is the first report of the complete mito-genome for Phyllocephalinae.

The complete mitogenome of *G. affinis* is a 16,011 bp long double-stranded circular molecule (GenBank accession no. MG182695), with a high A + T nucleotide content (45.89% A, 32.53% T, 12.06% C and 9.52% G); it is similar to other reported hemipteran mitogenomes (Hua et al. 2008; Zhao et al. 2017b).

It contains13 protein-coding genes (PCGs), 22 transfer RNA genes (tRNAs), two rRNA unit genes (rrnL and rrnS) and a non-coding A + T-rich region. The gene order and orientation of the mitochondrial genes are identical to those of most other true bugs (Hua et al. 2009; Zhao et al. 2017a, 2017b), which is considered to be ancestral arrangement.

Most PCGs share the start codons of ATN (ATT for *ND2*, *ATP8*, *ND5*, *ND4L* and *ND6*; ATG for *ATP6*, *COX III*, *ND4*, *CYTB* and *ND1*; ATA for *COX II* and *ND3*), except that *COI* starts with TTG. This unconventional start codon has also been reported for some other heteropterans (Hua et al. 2008; Zhao et al. 2017a, 2017b). All PCGs share the same termination codon TAA.

The 22 tRNAs range from 64 to 73 bp, and all have a typical cloverleaf secondary structure except trnS-*Ser* ^(AGN)^, which lacks a dihydrouridine (DHU) arm. rrnL is 1290 bp long with an A + T content of 78.29%, while rrnS is 802 bp long with an A + T content of 78.55%; they are separated from each other by trnV-*Val*. The D loop region (total length 1311 bp) is located between the 12S rRNA and trnI-*Ile*.

Phylogenetic analyses of the mitogenomes of *G. affinis* and other Pentatomoidea species were conducted using Bayesian inference (BI) with 17 nucleotide sequences of 13 mitochondrial PCGs (Zhou et al. 2011). The results confirmed the monophyly of each family of Pentatomoidea with high node support; Asopinae and Podopinae formed a sister group (Figure 1). The species *G. affinis*, which represents the subfamily Phyllocephalinae, was clustered with one species of Pentatominae and separated from other two subfamilies. This suggested that Pentatominae might not be monophyletic or Phyllocephalinae was more closely related to Pentatominae. This is the first sequenced complete mito-genome from the subfamily Phyllocephalinae. The mitogenomic data of *G. affinis* will help to better understand the population genetics and evolution of Pentatomoidea.

**Figure 1.**
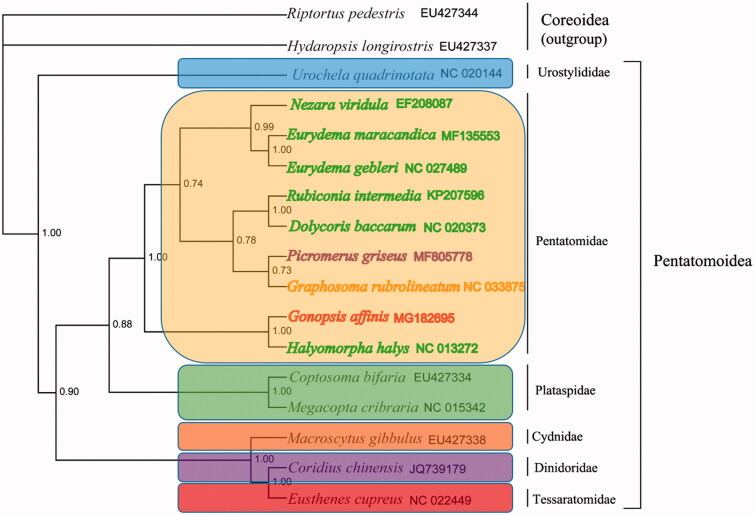
Phylogenetic relationship of *Gonopsis affinis* within Pentatomoidea inferred from 13 protein-coding genes (PCGs). Numbers on branches are Bayesian posterior probabilities. Families are delimited by coloured rectangles, and the four subfamilies of Pentatomidae are indicated by coloured species names.
